# Gut Microbiome and Transcriptomic Changes in Cigarette Smoke-Exposed Mice Compared to COPD and CD Patient Datasets

**DOI:** 10.3390/ijms25074058

**Published:** 2024-04-05

**Authors:** Lei Wang, Pim J. Koelink, Johan Garssen, Gert Folkerts, Paul A. J. Henricks, Saskia Braber

**Affiliations:** 1 Division of Pharmacology, Utrecht Institute for Pharmaceutical Sciences, Faculty of Science, Utrecht University, 3584 CG Utrecht, The Netherlands; l.wang@uu.nl (L.W.); j.garssen@uu.nl (J.G.); g.folkerts@uu.nl (G.F.); p.a.j.henricks@uu.nl (P.A.J.H.); 2 Department of Pathology and Medical Biology, University Medical Center Groningen, University of Groningen, 9713 GZ Groningen, The Netherlands; 3 Tytgat Institute for Liver and Intestinal Research, Amsterdam University Medical Centers, Amsterdam Gastroenterology, Endocrinology, Metabolism (AGEM), 1105 BK Amsterdam, The Netherlands; p.j.koelink@amsterdamumc.nl; 4 Nutricia Research, 3584 CT Utrecht, The Netherlands

**Keywords:** COPD, cigarette smoke, gut–lung axis, IBD, gut microbiome, transcriptome, gene expression profiling

## Abstract

Chronic obstructive pulmonary disease (COPD) patients and smokers have a higher incidence of intestinal disorders. The aim of this study was to gain insight into the transcriptomic changes in the lungs and intestines, and the fecal microbial composition after cigarette smoke exposure. Mice were exposed to cigarette smoke and their lung and ileum tissues were analyzed by RNA sequencing. The top 15 differentially expressed genes were investigated in publicly available gene expression datasets of COPD and Crohn’s disease (CD) patients. The murine microbiota composition was determined by 16S rRNA sequencing. Increased expression of MMP12, GPNMB, CTSK, CD68, SPP1, CCL22, and ITGAX was found in the lungs of cigarette smoke-exposed mice and COPD patients. Changes in the intestinal expression of CD79B, PAX5, and FCRLA were observed in the ileum of cigarette smoke-exposed mice and CD patients. Furthermore, inflammatory cytokine profiles and adhesion molecules in both the lungs and intestines of cigarette smoke-exposed mice were profoundly changed. An altered intestinal microbiota composition and a reduction in bacterial diversity was observed in cigarette smoke-exposed mice. Altered gene expression in the murine lung was detected after cigarette smoke exposure, which might simulate COPD-like alterations. The transcriptomic changes in the intestine of cigarette smoke-exposed mice had some similarities with those of CD patients and were associated with changes in the intestinal microbiome. Future research could benefit from investigating the specific mechanisms underlying the observed gene expression changes due to cigarette smoke exposure, focusing on identifying potential therapeutic targets for COPD and CD.

## 1. Introduction

Chronic obstructive pulmonary disease (COPD) was the third leading global cause of death in 2019, and is caused by exposure to harmful gases and particles; genetic factors may also contribute to the development of COPD [1]. COPD is associated with inflammatory bowel disease (IBD) [2], and COPD patients have a higher incidence and prevalence of IBD [3]. Cigarette smoke is composed of a complex mixture of over 4500 chemicals including harmful agents, such as carbon monoxide, nicotine, oxidants, fine particulate matter, and aldehydes. These components are considered some of the most detrimental factors for COPD pathogenesis [4,5]. The impact of cigarette smoke exposure on the respiratory tract has been widely studied. Recently, attention has been drawn to cigarette smoke-induced changes within the gastrointestinal tract [6]. One explanation for the lung–gut interaction following cigarette smoke exposure might be related to lung-induced systemic inflammation. In addition, cigarette smoke particles might enter the gastrointestinal tract due to mucociliary clearance of the lung or direct swallowing [7]. Recent studies have unveiled a complex neural connection between the lung and gut axis, suggesting that the interactions between respiratory health and gastrointestinal microbiota are also mediated through intricate neural pathways [8,9,10]. Although our group published a review about the advances in understanding the bidirectional crosstalk between the gut and the lungs in COPD [11], the underlying mechanisms have not yet been fully clarified.

The importance of the gut–lung axis has been observed in the development of COPD, and changes in the gut may potentiate inflammation and the progression of COPD [12]. The intestine is the largest immune organ in the body, containing around 70% of the host’s immune cells [13]. It also harbors various commensal microorganisms that are crucial for the functioning of the mucosal immune system [14]. Due to the vital role of the intestine in both health and disease, it is essential to better understand the intestinal responses induced after exposure to cigarette smoke. An increased knowledge of the molecular mechanisms underlying the immune responses in COPD patients could contribute to better therapeutic management of COPD and COPD-related intestinal comorbidities.

Recent advances in genomics have enabled genome-wide mRNA profiling, a valuable tool in identifying host immune responses and associated gene regulatory networks [15]. To gain insight into the lung–gut axis in COPD, the molecular changes in the lung and ileum (distal small intestine) were investigated in a murine model of cigarette smoke-induced COPD through KEGG pathway analysis. The highly differentially expressed genes in the murine lung were compared with the altered gene expression profile in the lungs of COPD patients, and the highly differentially expressed genes in the ileum of cigarette smoke-exposed mice were compared with the gene expression changes in the ileum of Crohn’s disease (CD) patients. In addition, fecal microbiome profiles in cigarette smoke-exposed mice were assessed by 16S rRNA sequencing.

## 2. Results

### 2.1. Differential Changes in Lung Tissue Transcriptome of Cigarette Smoke-Exposed Mice

**Figure 1:**
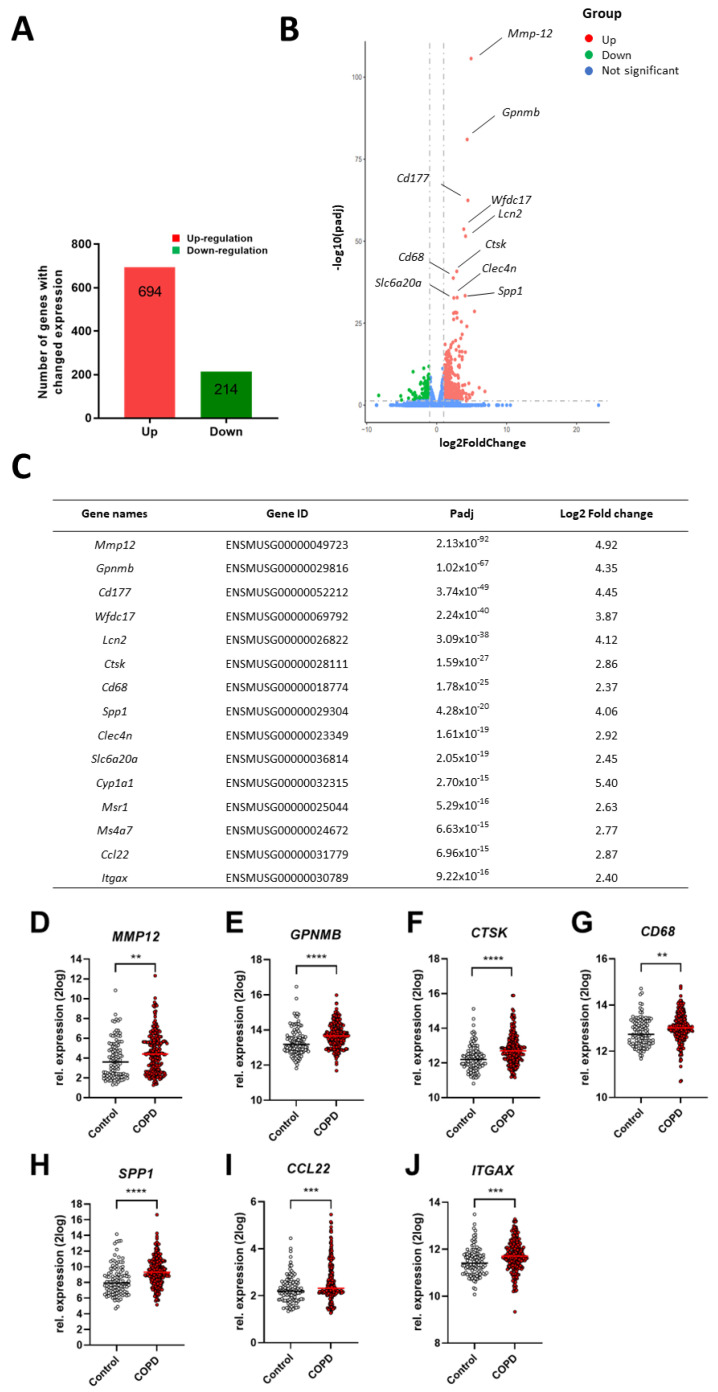
Differential changes in lung tissue transcriptome of cigarette smoke-exposed mice and overlapping transcriptome changes in COPD patients. Mice were exposed to air or cigarette smoke for 72 days and RNA sequence analysis of their lung tissues was performed. The number of significantly up-regulated (red) and down-regulated (green) genes (**A**) and a volcano plot with these differentially expressed genes (**B**) are depicted. Red dots represent the up-regulated genes, green dots represent down-regulated genes, while blue dots indicate the non-significantly altered genes with a padj < 0.05 and |log2(Fold Change)| > 1. The top 15 up- or down-regulated genes (padj < 0.05 and |log2(Fold Change)| > 1) in the lung tissues of cigarette smoke-exposed mice are depicted in Table (**C**). *n* = 3 mice/group. The top 15 up- or down-regulated genes in the murine lungs were also examined in COPD patients. MMP12 (**D**), GPNMB (**E**), CTSK (**F**), CD68 (**G**), SPP1 (**H**), CCL22 (**I**), and ITGAX (**J**) gene expression levels from COPD patients were compared to those of healthy individuals; *n* = 220 for COPD patients, *n* = 108 for healthy individuals. Values are expressed as mean  ±  SEM. ** *p* < 0.01, *** *p* < 0.001, **** *p* < 0.0001 for COPD group compared with control group.

To investigate the influence of cigarette smoke exposure on gene expression in murine lung tissues, mice were exposed to air or cigarette smoke for 72 days, and RNA-sequence analysis was performed on whole lung tissues. We identified 908 differentially expressed genes in the lung (padj < 0.05, |log2 (Fold Change)|> 1), of which, 694 genes were up-regulated and 214 genes were down-regulated in cigarette smoke-exposed animals (Figure 1A). The differentially expressed genes in response to cigarette smoke are shown in the volcano plot in Figure 1B, with red dots representing the up-regulated genes and green dots representing the down-regulated genes. The top 15 most significantly changed genes upon cigarette smoke exposure were all up-regulated in cigarette smoke-exposed mice and are depicted in Figure 1C.

### 2.2. Comparison of Lung Gene Expression Profiles of Cigarette Smoke Exposed-Mice and COPD Patients

To gain insight into the translational potential of the murine model of cigarette smoke-induced COPD, the transcriptomic profile of lung tissues from cigarette smoke-exposed mice was compared with the transcriptomic changes in the lungs of COPD patients. Lung tissue gene expression datasets were obtained from the Gene Expression Omnibus (GEO). Of the top 15 most significantly up-regulated genes upon cigarette smoke exposure in the murine model, 7 were also found to be significantly up-regulated in the lungs of COPD patients (MMP12, SPP1, CCL22, ITGAX, GPNMB, CTSK, and CD68; Figure 1D–J), 5 genes were not significantly elevated (CD177, LCN2, SLC6A20, CYP1A1, and MSR1; Supplementary Figure S1), while the 3 remaining genes (Wfcd17, Clec4n, and Ms4a7) or their human orthologs were not present in the database. This indicates that this murine model of cigarette smoke-induced COPD shows similarities with the development of COPD in humans.

### 2.3. Differential Changes in Ileum Transcriptome of Cigarette Smoke-Exposed Mice

**Figure 2:**
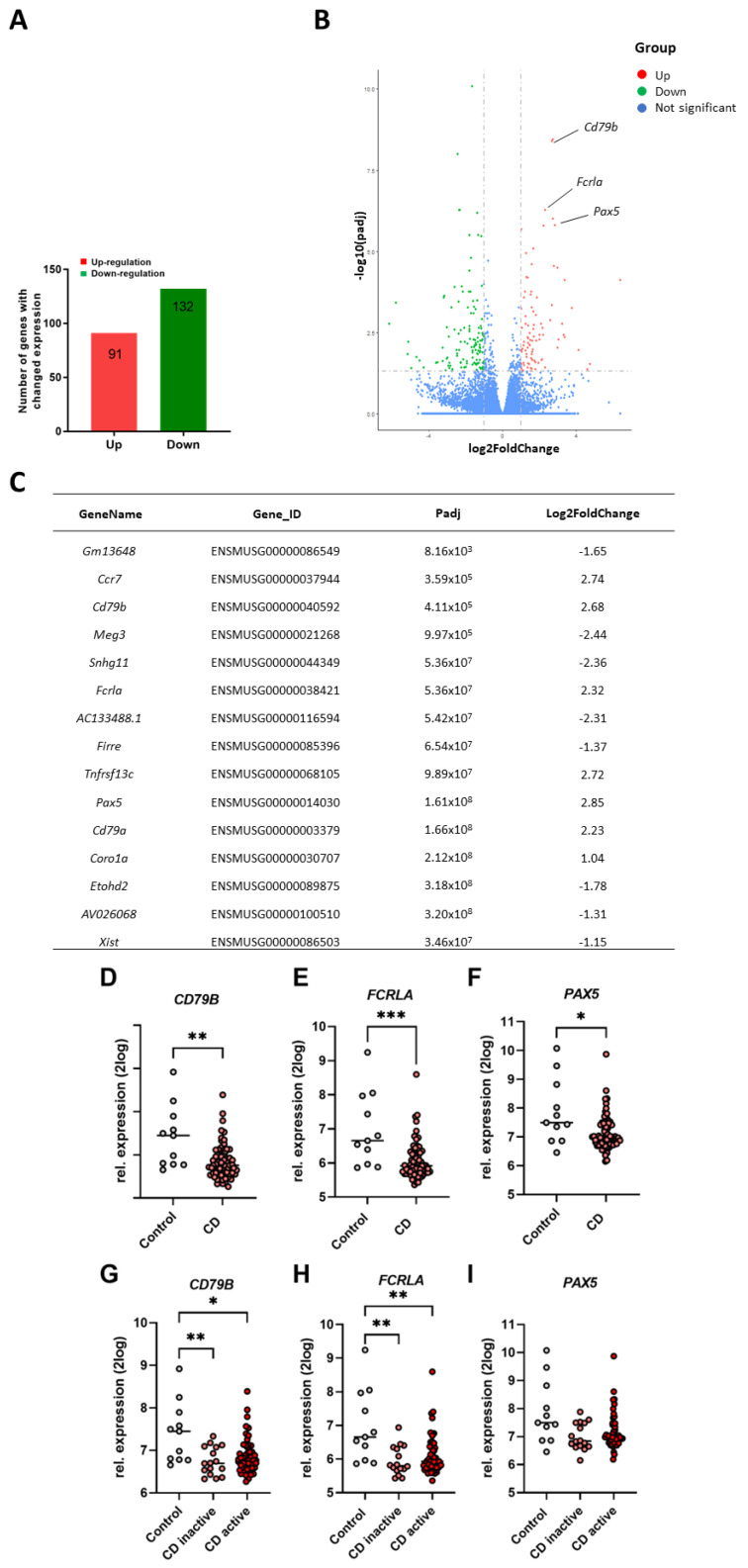
Differential changes in ileum transcriptome of cigarette smoke-exposed mice and overlapping transcriptome changes in CD patients. Mice were exposed to air or cigarette smoke for 72 days and RNA-sequence analysis of ileum was performed. The number of significantly up-regulated (red) and down-regulated (green) genes (**A**) and a volcano plot with these differentially expressed genes (**B**) are depicted. Red dots represent the up-regulated genes, green dots represent down-regulated genes, while blue dots indicate the non-significantly altered genes with a padj < 0.05 and |log2(Fold Change)| > 1. The top 15 up- or down-regulated genes (padj < 0.05 and |log2(Fold Change)| > 1) in ileum of cigarette smoke-exposed murine lungs are depicted in Table (**C**). *n* = 5 mice/group. The top 15 up- or down-regulated genes in murine ileum were also examined in the CD patients. CD79B (**D**), FCRLA (**E**) and PAX5 (**F**) gene expression levels in ileum of CD patients were compared to healthy individuals; CD79B (**G**), FCRLA (**H**) and PAX5 (**I**) gene expression levels in ileum of inactive and active CD patients were compared to healthy individuals. *n* = 67 for CD patients (*n* = 16 for inactive CD and *n* = 51 for active CD patients), *n* = 11 for healthy individuals. Values are expressed as mean  ±  SEM. * *p* < 0.05, ** *p* < 0.01, *** *p* < 0.001, CD group compared with control group. ** *p* < 0.01, CD inactive group compared with control group. * *p* < 0.05, ** *p* < 0.01, CD active group compared with control group.

To investigate the effect of cigarette smoke exposure on gene expression in murine intestinal tissues, an RNA sequence analysis was performed on whole proximal (duodenum) and distal (ileum) small intestinal tissues. A total of 223 genes were differentially expressed in the ileum of cigarette smoke-exposed mice compared to air-exposed mice (padj < 0.05, |log2 (Fold Change)| > 1), with up-regulated 91 genes and 132 down-regulated genes (Figure 2A). The differentially expressed genes in response to cigarette smoke are shown in the volcano plot in Figure 2B, with red dots representing the up-regulated genes and green dots representing the down-regulated genes. The top 15 differentially expressed genes are depicted as a table in Figure 2C.

In addition, 94 genes were differentially expressed (padj < 0.05, |log2 (Fold Change)| > 1) with 84 genes up-regulated and 10 genes down-regulated in the duodenum of cigarette smoke-exposed mice, as depicted in Supplementary Figure S2A. The differentially expressed genes in response to cigarette smoke are shown in a volcano plot (Supplementary Figure S2B). 18 common genes were differentially expressed in both duodenum and ileum of cigarette smoke-exposed mice.

### 2.4. Comparison of Murine Ileum Gene Expression Profiles versus Ileum Gene Expression Levels of CD Patients

To clarify whether the transcriptomic changes in the intestine observed in the murine model of cigarette smoke-induced COPD are similar to IBD-like intestinal changes, the murine transcriptomic profile was compared to the transcriptomic changes found in the ileum of CD patients. Of the top 15 differentially expressed genes in the murine ileum, 3 genes (CD79B, FCRLA, and PAX5) were differentially expressed in the ileum of patients with CD (Figure 2D–F), while 5 did not show an altered expression (CD79A, MEG3, CORO1A, TNFRSF13C, and CCR7; Supplementary Figure S3). The seven remaining genes (Gm13648, Snhg11, Fcrla, AC133488.1, Firre, Etohd2, AV026068, and Xist) or their human orthologs were not present in the database. Surprisingly, CD79B, FCRLA, and PAX5 were all up-regulated by cigarette smoke exposure in the murine model, while they were down-regulated in CD, especially in actively inflamed tissues (Figure 2G–I).

### 2.5. Overlapping Pathways in the Lung and Ileum of Cigarette Smoke-Exposed Mice

**Figure 3:**
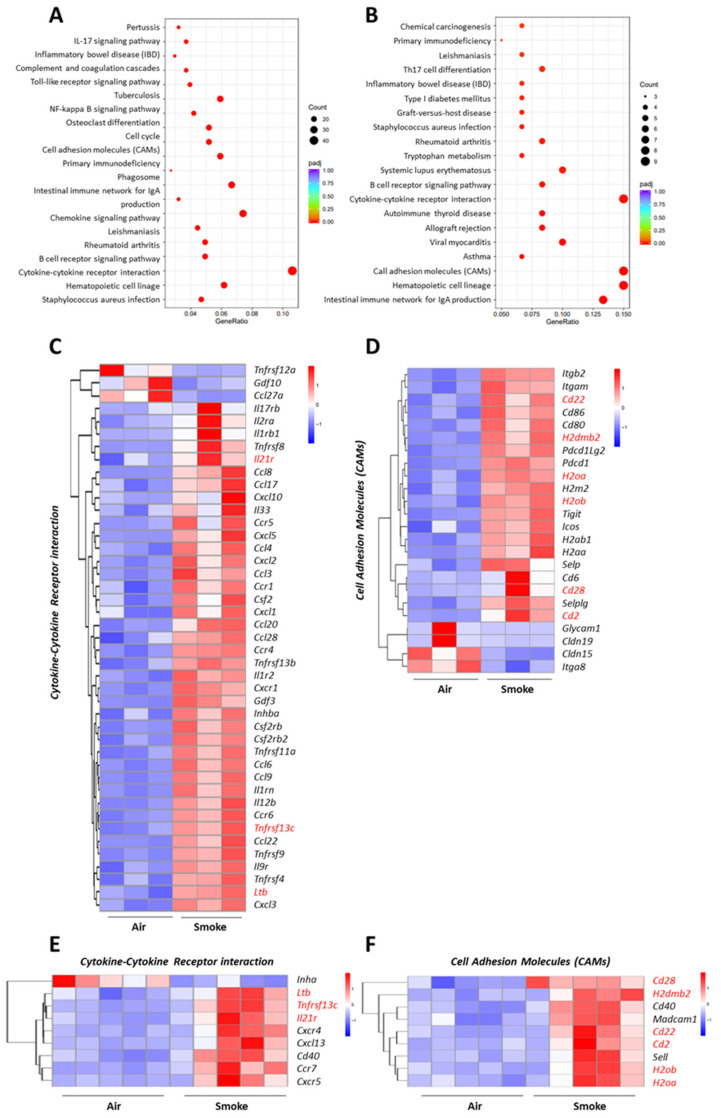
Overlapping pathways in the lungs and ileum of cigarette smoke-exposed mice. KEGG pathway enrichment analysis of differentially expressed genes in lung tissues (**A**) and ileum (**B**) of cigarette smoke-exposed mice are depicted as dot plots. The size of the dots represents the number (counts) of differentially expressed genes in that particular pathway, while the color of the dots represents the padj values (based on the scale bar). Heatmaps depicting the expression levels of genes enriched in the cytokine–cytokine receptor interaction pathway in the lungs (**C**) and ileum (**E**) or in the cell adhesion molecule pathway in the lungs (**D**) and ileum (**F**) of air- and cigarette smoke-exposed mice. *n* = 3 mice/group for lung tissues, *n* = 5 mice/group for the ileum samples.

To identify the signaling pathways that are associated in both lung and intestinal tissues upon cigarette smoke exposure, a KEGG pathway enrichment analysis was performed. The top 20 pathways (padj < 0.05, |log2 (Fold Change)| > 1) in the lung and in the ileum are shown in Figure 3A,B. The two signaling pathways that are affected by cigarette smoke exposure in both the lungs and intestines are the cytokine–cytokine receptor interaction and cell adhesion molecule pathways.

Heatmaps of the specific genes in the cytokine–cytokine receptor interaction pathway that are significantly altered in the lungs and intestines are depicted in Figure 3C,E. An up-regulation of Il21r, Tnfrsf13c, and Ltb from the cytokine–cytokine receptor interaction pathway by cigarette smoke (marked in red) was found in both organs.

For the cell adhesion molecule pathway, the heatmaps of the significantly altered genes in the lungs and intestines are shown in Figure 3D,F. Cd28, H2dmb2, Cd22, Cd2, H2ob, and H2oa were up-regulated by cigarette smoke exposure (depicted in red).

In addition, the cytokine–cytokine receptor interaction pathway was also the most enriched pathway in the duodenum (proximal small intestine) of cigarette smoke-exposed mice (Supplementary Figure S2C). The heatmap shows that Il21r levels were increased by cigarette smoke exposure in both intestinal parts and lung tissues (Figure 3E and Supplementary Figure S2D).

### 2.6. Altered Fecal Microbial Composition in Cigarette Smoke-Exposed Mice

**Figure 4:**
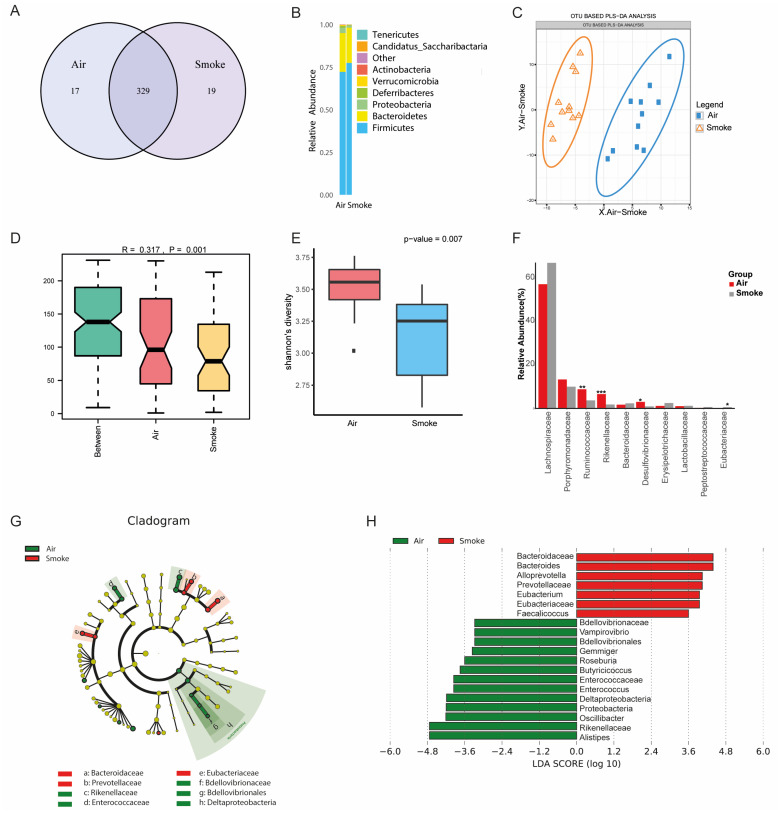
Fecal microbial composition in cigarette smoke-exposed mice. Mice were exposed to cigarette smoke for 72 days and fecal samples were collected to measure the microbial composition. Operational taxonomic unit (OTU) Venn diagrams of shared and unique OTUs in the feces of cigarette smoke- and air-exposed mice (**A**). Stacked column plots of the most abundant bacterial populations in the feces of cigarette smoke- and air-exposed mice (**B**). OTU-based partial least squares discriminant analysis (PLS-DA) (**C**), ANOSIM similarity analysis (**D**), and Shannon index of alpha diversity (**E**). The top 10 families were chosen to show the average relative abundance of smoke and air exposure groups (* *p* < 0.05, ** *p* < 0.01, *** *p* < 0.001) (**F**). Cladogram of linear discriminant analysis (LDA) effect size (LEfSe) analysis of microbial abundance from phylum to genus level (**G**); LDA scores of the degree of differentiation between air- and cigarette smoke-exposed mice (**H**).

To investigate the effect of smoke exposure on the murine microbiota composition, fecal samples were collected and subjected to taxonomic profiling using 16S rRNA sequencing to determine microbiota community composition. We identified 365 operational taxonomic units (OTUs), of which, 329 were found in the feces of both cigarette smoke- and air-exposed mice, 19 were unique in smoke-exposed mice, and 17 were unique in air-exposed mice (Figure 4A). The core microbiota was dominated by the phyla Firmicutes, Bacteroidetes, and Proteobacteria in both the smoke- and air-exposed groups (Figure 4B). An OTU-based partial least squares discriminant analysis (PLS-DA) demonstrated a clear separation of the microbiome communities of the air- and smoke-exposed mice (Figure 4C). An ANOSIM similarity analysis indicated that the differences between the groups were significantly larger than the differences within the groups (Figure 4D). The richness and evenness of the gut microbial taxa, as measured using the Shannon index of alpha diversity, showed a significant reduction in the cigarette smoke-exposed mice (*p* = 0.007, Figure 4E). In cigarette smoke-exposed mice, Eubacteriaceae was enriched, while Ruminococcaceae, Desulfovibrionaceae, and Rikenellacease were depleted in the fecal samples of the cigarette smoke-exposed mice (see Figure 4F). The linear discriminant analysis effect size algorithm (LEfSe) analysis results further showed significantly different signatures between the air- and smoke-exposed mice (Figure 4F,G). Using an LDA analysis, we found an increase in Bacteroidaceae, Bacteroides, Alloprevotella, Prevotellaea, Eubacterium, Eubacteriaceae, and Faecalicoccus in the cigarette smoke-exposed mice (Figure 4H).

## 3. Discussion

Cigarette smoke is one of the major triggers of lung inflammation and respiratory diseases, such as COPD [16]. Intestinal diseases are commonly observed in COPD patients [17] and the incidence of IBD is higher in COPD patients compared to healthy subjects [18]. Nowadays, intestinal immune responses induced by cigarette smoke are receiving more and more attention [19]. In our previous study, we demonstrated changes in intestinal homeostasis and immunity in a cigarette smoke- and LPS-induced murine model for COPD [7]. The impact of smoking on the gut microbiome is multifaceted, involving several direct and indirect mechanisms, like (1) systemic inflammation, (2) direct exposure to cigarette smoke components via swallowing, and (3) systemic hypoxia. The exact pathogenesis of these observations is not yet clear. Therefore, we discussed the postulated underlying mechanisms of the gut–lung crosstalk in COPD in a recent review paper [11] and investigated the intestinal microbial composition, molecular changes, and associated signaling pathways in the lungs and intestines of mice exposed to cigarette smoke in the current study. The changes in microbiota alongside the gene sequencing of the intestines throughout the duration of smoke exposure have not been thoroughly explored in the existing literature. This novel angle offers deeper insights into the genetic mechanisms underlying the impact of cigarette smoke on gut health, providing a more comprehensive understanding of the interaction between smoke exposure and intestinal changes.

Pre-clinical animal models are a valuable tool for understanding the pathogenesis of COPD and its related comorbidities. To gain insight into the translational potential of a murine model of cigarette smoke-induced COPD, we compared the transcriptional changes in mice with the transcriptomic profiles of the lungs of COPD patients. Seventy-two days of cigarette smoke exposure induced the differential expression of 908 genes in the lungs compared with air-exposed mice. Among the top 15 differentially expressed (up-regulated) genes in lungs of cigarette smoke-exposed mice, MMP12, GPNMB, CTSK, CD68, SPP1, CCL22, and ITGAX were also significantly up-regulated in the lung tissues of COPD patients. In another study, Mmp12, GPNMB, CTSK, and CD68 were identified among the top 20 genes that were up-regulated in murine lung tissues after 8–16 and 24 weeks of cigarette smoke exposure [20].

MMP-12 belongs to the matrix metalloproteinases family, which contributes to the remodeling of the small airways and to the proteolytic degradation of the alveolar wall matrix, leading to emphysema [21]. GPNMB, an endogenous glycoprotein, can influence the pathogenesis of COPD, for instance, through its contribution to tissue remodeling in COPD by promoting the secretion of MMP-9, another matrix metalloproteinase [22]. CTSK is a cysteine protease that is stimulated by inflammatory cytokines and released after tissue injury, contributing to the destruction of connective tissues in the lungs [23]. CTSK is up-regulated in alveolar macrophages of emphysema patients and in the lungs of cigarette smoke-exposed guinea pigs. CTSK is partially responsible for the loss of lung elasticity and recoil observed during the development of emphysema [23,24]. The expression of SPP1, Secreted Phosphoprotein 1, positively correlates with COPD severity, as assessed by forced expiratory volume in 1 s (FEV1) measurements [25]. In addition, SPP1 was the only differentially expressed gene that was up-regulated in both patients with COPD and lung cancer. This indicates that the up-regulation of SPP1 in COPD might be associated with the increased risk of lung cancer in these patients [26]. CCL22, chemokine (C-C motif) ligand 2, is a monocyte-derived chemokine and exerts functions in multiple lung-related diseases [27]. CCL22 is elevated in bronchial tissues from COPD patients and could potentially affect adaptive immune responses in COPD disease progression [28,29]. ITGAX, the gene that encodes CD11c (a leukocyte integrin), mediates the adherence of neutrophils and monocytes to activated endothelial cells [30]. ITGAX was identified as one of the top seven genes, which is increased by  >2.5-fold in emphysema patients [31]. However, information regarding the role of ITGAX in the development of COPD is scarce.

The current study showed that 72 days of cigarette smoke exposure induced altered gene profiles in murine lungs that are comparable to the changes observed in COPD patients, confirming the COPD-like features in our murine model. Besides the changes observed in the expression of Mmp12, Gpnmb, Ctsk, Cd68, Spp1, and Ccl22 in both murine lung tissues and lung tissues of COPD patients, other COPD characteristics, such as an enlarged mean linear intercept (Lm) and increased number of neutrophils and macrophages in the bronchoalveolar lavage fluid, were also observed in this murine model of cigarette smoke-induced COPD [32].

Having determined that the mice exposed to 72 days of cigarette smoke show characteristics of COPD patients, we focused on the intestinal gene expression alterations upon cigarette smoke exposure. Ten weeks of cigarette smoke exposure induced more alterations in gene expression levels in the distal small intestine (223 altered genes; padj < 0.05, |log2(Fold Change)| > 1)) compared to the proximal small intestine (94 altered genes). We compared the changes in the distal small intestine to the changes found in the ileum of patients with CD. CD, one of the major forms of IBD, can affect all segments of the gastrointestinal tract, most commonly the terminal ileum [33]. Smoking is known to be associated with the development of CD, and particularly impacts ileum homeostasis and inflammatory processes [34]. To clarify whether the transcriptomic changes in the intestine observed in the murine model of cigarette smoke-induced COPD are comparable to IBD-like intestinal changes, the altered murine genes were evaluated in the ileum of CD patients. From the top 15 differentially expressed genes observed in the ileum of cigarette smoke-exposed mice, CD79B, PAX5, and FCRLA were up-regulated in the murine ileum and down-regulated in CD patients. Interestingly, all three genes are involved in the functioning of B cells, a critical cell type in immune homeostasis at mucosal surfaces, including the gastrointestinal tract. B cell-related abnormalities, such as lympho-plasmacytic infiltrates and anti-microbial antibodies, have been reported in IBD patients [35]. CD79B is a B cell lineage-specific gene that is necessary for the expression and function of the B cell antigen receptor. The PAX5 gene encodes a B cell lineage-specific activator protein that is expressed at early stages of B cell differentiation, and the FCRLA gene encodes a protein that is selectively expressed in B cells and possibly involved in B cell development, but the exact role of these genes in IBD still needs to be determined. Targeting B cell-related genes and receptor pathways may hold promise as novel therapies for IBD or for the intestinal symptoms observed in COPD patients [35].

In the current study, the changes observed in the ileum of cigarette smoke-exposed mice were not in agreement with the changes observed in the ileum of CD patients, indicating that there were no obvious IBD-like abnormalities in this model. However, this could be time-dependent and studies with long-term exposure to cigarette smoke might lead to more IBD-like changes.

Subsequently, the potential link between the gut and lungs in our cigarette smoke-induced COPD model was investigated by performing KEGG pathway enrichment analyses in both organs (padj < 0.05, |log2(Fold change)| > 1). The genes in both the lung and intestinal tissues that were affected by cigarette smoke exposure mainly belonged to the cytokine–cytokine receptor interaction pathway and pathways related to cell adhesion molecules. Il21r, Tnfrsf13c, and Ltb were up-regulated by cigarette smoke exposure and are related to the cytokine–cytokine receptor interaction pathway; Cd28, H2dmb2, Cd22, Cd2, H2ob, and H2oa were also up-regulated by cigarette smoke exposure and are associated with the cell adhesion molecule pathway in both the lungs and intestines. COPD is strongly associated with systemic alterations including altered levels of circulating cytokines and adhesion molecules [36]. There are several therapeutic approaches that target the cytokine-mediated inflammation in COPD. However, inhibiting specific cytokines may not provide sufficient clinical benefits [37]. Considering the complex interactions between various cytokines involved in inflammatory diseases [38], broad-spectrum anti-inflammatory approaches or targeting multiple cytokines could be considered as an approach for COPD treatment. Cell adhesion molecules play a critical role in the recruitment and migration of cells to the sites of inflammation in patients with COPD [39]. Although the up-regulated genes, including Il21r, Tnfrsf13, Ltb, Cd28, H2dmb2, Cd22, Cd2, H2ob, and H2oa, related to the common pathways observed in the lungs and ileum (Figure 3C–F) have not been proven to play a role in the interactions between the gut and lungs, some of these genes, such as Il21, have been shown to play important roles in chronic inflammatory diseases [40,41].

The Il21r gene is related to the cytokine-cytokine interaction pathway and was significantly up-regulated in the lungs and the proximal and distal small intestine in cigarette smoke-exposed mice. IL21R transduces the growth-promoting signal of IL21, and is important for the proliferation and differentiation of T cells, B cells, and natural killer cells. The IL21/IL21R interaction plays an important role in a variety of inflammatory diseases [40]. IL21R has been considered as a marker for Th17 cells; however, Th17 cells have not yet been specifically identified in the lungs of COPD patients, but Th17-related cytokines have been observed in the bronchial mucosa [42]. Duan et al. observed that the levels of IL21 and the frequencies of Th1, Tc1, CD4+ IL21+, CD4+ IL21R+, and CD8+ IL21R+ T cells were much higher in CS-exposed mice compared to control [43]. In addition, IL21 produced by CD4+ T cells could promote a Th1/Tc1 response, leading to systemic inflammation in emphysema [43]. Recent studies indicated that the level of IL21 was significantly increased in peripheral blood and intestinal tissues of patients with CD or UC, suggesting that IL21/IL21R signaling may be involved in the pathogenesis of IBD [41]. Therefore, IL21/IL21R might be an interesting target for future research related to exploring the lung–gut crosstalk.

Besides the involvement of the immune system in the pathogenesis of COPD, COPD patients have an altered gut microbiota compared with healthy individuals and cigarette smoking is associated with intestinal microbiota dysbiosis [44]. In the current study, an altered microbiota composition and a reduction in bacterial diversity was observed in cigarette smoke-exposed mice compared to air-exposed mice. Interestingly, both COPD patients and IBD patients have reduced species richness, and imbalances in families, classes, and phyla relative to healthy volunteers [3]. In cigarette smoke-exposed mice, *Eubacteriaceae* was enriched, while *Ruminococcaceae, Desulfovibrionaceae,* and *Rikenellacease* were depleted in the fecal samples of the cigarette smoke-exposed mice, which is generally in line with the intestinal microbiome of COPD patients [45,46]. An increased abundance of *Eubacteriaceae* was found in COPD patients, and *Eubacterium rectale* can contribute to colorectal cancer initiation via promoting colitis [47]. The *Ruminococcaceae* families are well-known short-chain fatty acid (SCFA) producers. SCFAs, such as acetate, propionate, and butyrate, are derived from intestinal microbial fermentation of indigestible foods and have been described as important metabolites in maintaining intestinal homeostasis [48]. They are known to strengthen the gut barrier function and modulate immune functions [49]. Both COPD and IBD patients have lower levels of SCFAs compared to healthy individuals [44,48]. The *Desulfovibrionaceae* family was also depleted in COPD patients; however, the data were highly variable between individuals [45]. The abundance of *Desulfovibrionaceae* was increased in UC patients [50]. The *Rikenellaceae* genus was also less prevalent in COPD patients than in healthy controls [45]. However, *Rikenellaceae* was more abundant in irritable bowel syndrome (IBS) than IBD, and was underrepresented in IBD [51]. All these findings show that there are some similarities in the gut microbiota dysbiosis of COPD and IBD patients. The LEfSe analysis results further showed significantly different signatures between the air- and smoke-exposed mice. Interestingly, mice that received a fecal transplant from COPD patients that presented a *Prevotella*-dominated gut microbiome with lower levels of SCFAs developed lung inflammation and, upon subsequent cigarette smoke exposure, showed an aggravated deterioration of lung function [44]. In the current study, an increase in *Prevotellaea* was also detected in the fecal samples of the cigarette smoke-exposed mice.

In summary, 72 days of cigarette smoke exposure altered gene expression in the murine lung tissue. Some of these genes are also affected in COPD patients, which confirms the COPD-like changes in this murine model of cigarette smoke-induced COPD. Cigarette smoke exposure significantly altered the expression of genes in the murine ileum; however, these changes were markedly different from the altered genes observed in the ileum of CD patients.. In addition, changes in the fecal microbiota composition and reduced bacterial diversity were observed in cigarette smoke-exposed mice. The cytokine–cytokine receptor interaction pathway and pathway related to cell adhesion molecules were the most enriched pathways observed in lungs as well as in the ileum, while the cytokine–cytokine receptor interaction pathway was also highly enriched in the duodenum.

In conclusion, our study not only reaffirms the impact of cigarette smoke on the gut microbiota but also pioneers investigations into the associated genetic changes in the intestine. These findings pave the way for future research aimed at uncovering the molecular mechanisms behind smoke-related gut alterations, for instance, by focusing on the most significantly altered genes and highly enriched overlapping pathways. Ultimately, this may contribute to the development of targeted therapies and public health strategies to mitigate these effects. These may include personalized dietary recommendations, such as incorporating interventions like probiotics, prebiotics, and dietary fiber to restore gut microbiota balance for conditions like COPD and related gastrointestinal comorbidities such as IBD.

## 4. Materials and Methods

### 4.1. Animals

Specific-pathogen free female Balb/c mice [52,53], 11–13 weeks old, were obtained from Charles River Laboratories. The mice were housed in filter-topped makrolon cages (Tecnilab-BMI, Someren, The Netherlands) with wood chip bedding (Tecnilab-BMI, Someren in The Netherlands) and tissues (VWR, Amsterdam, The Netherlands) were available as cage enrichment. The mice were kept under standard conditions on a 12 h light/dark cycle (lights on from 7.00 am to 7.00 pm) at a controlled relative humidity of 50–55% and temperature of 21 ± 2 °C at the animal facility of Utrecht University. Food (AIN-93M, SNIFF Spezialdiäten GmbH, Soest, Germany) and water were provided ad libitum and were refreshed once a week. The study described in this article is part of a larger trial, including an air control group, cigarette smoke exposure group, and 6 other groups [6]. In accordance with the purpose of this study, transcriptomic sequencing of lung and intestine samples of the air control and cigarette smoke exposure groups was performed. All animal procedures described in this study were approved by the Ethics Committee of Animal Research of Utrecht University, Utrecht, The Netherlands (AVD1080020184785), and were conducted in accordance with the governmental guidelines.

### 4.2. Cigarette Smoke Exposure

Mice in whole-body chambers were exposed to mainstream cigarette smoke or air by using a peristaltic pump (SCIQ 232, Watson-Marlow 323, Wilmington, MA, USA). A Plexiglas box containing four metal cages, each with four compartments, was used to expose the mice to either cigarette smoke or air. Two mice from the same home cage were placed in each compartment. Research cigarettes (3R4F) were obtained from the Tobacco Research Institute (University of Kentucky, Lexington, KY, USA) [54] and the filters were removed before use [52].

The mice were acclimatized to cigarette smoke exposure by gradually increasing the number of cigarettes during the first days of the experiment using 4 cigarettes (±10 min/day) on day 1; 6 cigarettes (±15 min/day) on day 2; 8 cigarettes (±20 min/day) on day 3; 10 cigarettes (±25 min/day) on day 4; 12 cigarettes (±30 min/day) on day 5; and 14 cigarettes (±35 min/day) from day 6 until the end of the study (day 72) [7]. The smoke chamber was connected to a peristaltic pump and vacuum to produce smoke and control the air circulation. The speed of the pump was kept at 35 rpm and the CO levels ranged between 200 and 400 ppm. The mass concentration of cigarette smoke total particulate matter (TPM) was determined by gravimetric analysis using type A/E glass fiber filter (PALL life sciences, Tijuana, Mexico). The TPM concentration in the smoke exposure box generated by 14 cigarettes reached approximately 828 μg/L (828 ± 4.5 μg/L) [52]. The mice were anesthetized by an intraperitoneal injection of ketamine/medetomidine (196.8 mg/kg and 1.32 mg/kg, respectively) approximately 18 h after the last air or smoke exposure, and lung and proximal and distal small intestinal tissues were isolated for the following measurements.

### 4.3. RNA Isolation and Gene Sequence

#### 4.3.1. RNA Preparation

Total RNA was isolated and extracted from tissues using the RNeasy Mini Kit according to the manufacturer’s protocol (Qiagen, Hilden, Germany). The RNA integrity and quantity were assessed using the RNA Nano 6000 Assay Kit of the Bioanalyzer 2100 system (Agilent Technologies, Santa Clara, CA, USA). RNA degradation and contamination were monitored on 1% agarose gels.

#### 4.3.2. Non-Directional Sequencing of Polyadenylated (polyA) mRNA

RNA samples were used for library preparation using an NEB Next^®^ Ultra RNA Library Prep Kit for Illumina^®^ Indices (New England Biolabs, MA, USA) to multiplex multiple samples. Briefly, mRNA was purified from total RNA using poly-T oligos attached to magnetic beads. After fragmentation, the first strand cDNA was synthesized using random hexamer primers followed by a second strand cDNA synthesis. The library was ready after end repair, A-tailing, adapter ligation, and size selection. After amplification and purification, the insert size of the library was validated on an Agilent 2100 and quantified using quantitative PCR (Q-PCR). Libraries were then sequenced on an Illumina NovaSeq 6000 S4 flowcell with PE150 according to results from library quality control and expected data volume. The library preparation/sequencing/analysis were performed by Novogene (UK) Company Limited (Cambridge, UK).

### 4.4. Clinical Data

Whole-transcriptome data were retrieved from Gene Expression Omnibus (GEO) datasets: GSE47460 (LGR consortium, lungs of COPD (*n* = 220) vs. controls (*n* = 108)) and GSE16879 (ilea of CD patients (*n* = 67) vs. healthy controls (*n* = 11)). The datasets were uploaded to and analyzed by the R2 Genomics Analysis and Visualization Platform (http://r2.amc.nl (accessed on 7 February 2024)).

### 4.5. DNA Extraction of Fecal Samples, Library Construction, Gut Microbiota Sequencing

Fecal samples were collected from individual mice on day 72 and stored at −80 °C until future use. Total DNA extraction and library construction were performed by BGI Genomics (Shenzhen, China). Total bacterial DNA was extracted using a MagPure Stool DNA KF Kit B (MD5115, Shenzhen, China) following the manufacturer’s instructions. The DNA was quantified with a Qubit Fluorometer (Invitrogen, Waltham, MA, USA) by using a Qubit dsDNA BR Assay Kit (Q32850, Invitrogen, USA) and the quality was checked by running an aliquot on a 1% agarose gel.

The variable V3-V4 regions of the bacterial 16S rRNA gene were amplified with degenerate PCR primers 341F (5′-ACTCCTACGGGAGGCAGCAG-3′) and 806R (5′-GGACTACHVGGGTWTCTAAT-3′). Both forward and reverse primers were tagged with Illumina adapter, pad, and linker sequences. PCR enrichment was performed in a 50 μL reaction containing 30 ng of the template, fusion PCR primers, and PCR master mix. The PCR cycling conditions were as follows: 94 °C for 3 min; 30 cycles of 94 °C for 30 s, 56 °C for 45 s, and 72 °C for 45 s; and final extension for 10 min at 72 °C. The PCR products were purified with AMPure XP beads (A63882, Beckman Coulter, Brea, CA, USA) and eluted using elution buffer. The libraries were qualified by an Agilent 2100 Bioanalyzer (Agilent, Santa Clara, CA, USA).

The validated libraries were used for sequencing by BGI (Shenzhen, China) on the Illumina HiSeq 2500 platform (Illumina, San Diego, CA, USA) following the standard pipelines of Illumina, generating 2 × 300 bp paired end reads with a coverage of 50k reads. To quantify the abundance of bacteria, the sequences were clustered into OTUs based on 97% sequence similarity. Species were classified into ‘others’ if their relative abundance was less than 0.5%.

### 4.6. Statistics

The gene sequencing data from the in vivo study were analyzed using Novosmart software (Novosmart; Cambridge, UK). The differential expression analysis between two groups was performed using the DESeq2 R package. The resulting *p*-values were adjusted using Benjamini and Hochberg’s approach for controlling the False Discovery Rate (FDR). Genes with an adjusted *p*-value < 0.05 found by DESeq2 were considered differentially expressed. KEGG pathway libraries were used to perform an enrichment analysis. Fecal microbial data from the in vivo study were analyzed using an online system (Dr. Tom) provided by BGI. Clinical data were analyzed by Mann–Whitney tests comparing two groups or Kruskal–Wallis tests followed by Dunn’s multiple comparisons tests, and were considered significant when *p* < 0.05.

## Data Availability

The raw data are stored at Utrecht University. The dataset used and/or analyzed during the current study is available from the corresponding author on reasonable request.

## Supplementary Materials

The supporting information can be downloaded at https://figshare.com/s/bfbaf37a5dd3d9e8fb62 (accessed on 4 February 2024).
